# Effect of a lung recruitment maneuver by high-frequency oscillatory ventilation in experimental acute lung injury on organ blood flow in pigs

**DOI:** 10.1186/cc4967

**Published:** 2006-07-12

**Authors:** Matthias David, Hendrik W Gervais, Jens Karmrodt, Arno L Depta, Oliver Kempski, Klaus Markstaller

**Affiliations:** 1 Department of Anesthesiology, Johannes Gutenberg-University, Mainz, Germany; 2 Institute of Neurosurgical Pathophysiology, Johannes Gutenberg-University, Mainz, Germany

## Abstract

**Introduction:**

The objective was to study the effects of a lung recruitment procedure by stepwise increases of mean airway pressure upon organ blood flow and hemodynamics during high-frequency oscillatory ventilation (HFOV) versus pressure-controlled ventilation (PCV) in experimental lung injury.

**Methods:**

Lung damage was induced by repeated lung lavages in seven anesthetized pigs (23–26 kg). In randomized order, HFOV and PCV were performed with a fixed sequence of mean airway pressure increases (20, 25, and 30 mbar every 30 minutes). The transpulmonary pressure, systemic hemodynamics, intracranial pressure, cerebral perfusion pressure, organ blood flow (fluorescent microspheres), arterial and mixed venous blood gases, and calculated pulmonary shunt were determined at each mean airway pressure setting.

**Results:**

The transpulmonary pressure increased during lung recruitment (HFOV, from 15 ± 3 mbar to 22 ± 2 mbar, *P *< 0.05; PCV, from 15 ± 3 mbar to 23 ± 2 mbar, *P *< 0.05), and high airway pressures resulted in elevated left ventricular end-diastolic pressure (HFOV, from 3 ± 1 mmHg to 6 ± 3 mmHg, *P *< 0.05; PCV, from 2 ± 1 mmHg to 7 ± 3 mmHg, *P *< 0.05), pulmonary artery occlusion pressure (HFOV, from 12 ± 2 mmHg to 16 ± 2 mmHg, *P *< 0.05; PCV, from 13 ± 2 mmHg to 15 ± 2 mmHg, *P *< 0.05), and intracranial pressure (HFOV, from 14 ± 2 mmHg to 16 ± 2 mmHg, *P *< 0.05; PCV, from 15 ± 3 mmHg to 17 ± 2 mmHg, *P *< 0.05). Simultaneously, the mean arterial pressure (HFOV, from 89 ± 7 mmHg to 79 ± 9 mmHg, *P *< 0.05; PCV, from 91 ± 8 mmHg to 81 ± 8 mmHg, *P *< 0.05), cardiac output (HFOV, from 3.9 ± 0.4 l/minute to 3.5 ± 0.3 l/minute, *P *< 0.05; PCV, from 3.8 ± 0.6 l/minute to 3.4 ± 0.3 l/minute, *P *< 0.05), and stroke volume (HFOV, from 32 ± 7 ml to 28 ± 5 ml, *P *< 0.05; PCV, from 31 ± 2 ml to 26 ± 4 ml, *P *< 0.05) decreased. Blood flows to the heart, brain, kidneys and jejunum were maintained. Oxygenation improved and the pulmonary shunt fraction decreased below 10% (HFOV, *P *< 0.05; PCV, *P *< 0.05). We detected no differences between HFOV and PCV at comparable transpulmonary pressures.

**Conclusion:**

A typical recruitment procedure at the initiation of HFOV improved oxygenation but also decreased systemic hemodynamics at high transpulmonary pressures when no changes of vasoactive drugs and fluid management were performed. Blood flow to the organs was not affected during lung recruitment. These effects were independent of the ventilator mode applied.

## Introduction

High-frequency oscillatory ventilation (HFOV) is a pressure-controlled, time-cycled method of mechanical ventilation in which a continuous distending pressure (CDP) expands the lung and superimposed pressure oscillations at high frequencies (4–15 Hz) from a coupled oscillator swing around the applied CDP. The pressure swings are significantly attenuated by the endotracheal tube and the respiratory system before reaching the alveolar level. The tidal volumes and pressure amplitudes at the alveolar level are therefore minimal. Active expiration by the superimposed pressure swings prevents air trapping [1]. HFOV theoretically has advantages such as the minimal applied tidal volumes at the alveolar level, avoiding volutrauma from tidal overdistension, whereas a constant high mean airway pressure (*P*_mean_) leads to lung recruitment over time [2].

A potential drawback to HFOV is the fact that spontaneous respiratory efforts must be suppressed. When similar *P*_mean _settings by HFOV or conventional ventilation are used, however, the amplitude of pressure and volume excursions is substantially different between both ventilatory modes. Despite the same arithmetic *P*_mean_, alveolar excursions occur around a greater gradient of pressures and volumes during conventional ventilation. It is well known that high airway pressures may lead to detrimental hemodynamic effects, mainly dependent on respiratory mechanics and the capacity of cardiovascular compensation [3,4]. Inspiratory lung inflation can alter the autonomic tone, pulmonary vascular resistance, ventricular filling by reduced venous return, and at high lung volumes, it interacts mechanically with the heart in the cardiac fossa to limit absolute cardiac volumes [3,4].

Current practice at the initiation of HFOV involves lung recruitment maneuvers, typically performed by increases of CDP in steps of 2–5 mbar up to 40 mbar [5-8]. Although increases of the CDP may improve oxygenation and gas exchange, the effects of high CDP and nearly constant lung volumes during HFOV upon organ blood flow have not been evaluated. The hemodynamics, transpulmonary pressure (*P*_T_), and organ blood flows were therefore measured in pigs with acute injured lungs during a sequence of similar *P*_mean _increases by HFOV and by conventional pressure-controlled ventilation (PCV). The primary objective of this study was to asses whether a recruitment procedure of the lung, at initiation of HFOV by stepwise increases of continuous distending pressures, impairs the hemodynamics and organ blood flow in lung-injured animals. Secondarily, we determined whether these effects are more pronounced during HFOV when compared with similar *P*_mean _settings in PCV.

## Materials and methods

### Animals and instrumentation

The study protocol was approved by the institutional and state animal care committee. Seven pigs (mean body weight, 26 kg; range, 23–27 kg) were anesthetized with fentanyl 0.005 mg/kg and thiopentone 10–15 mg/kg intravenously, followed by a continuous infusion of fentanyl (5 μg/kg/hour) and thiopentone (10 mg/kg/hour). Neuromuscular blockade was achieved with repeated intravenous bolus of pancuronium bromide (0.1 mg/kg). An adequate level of anesthesia was monitored clinically by observation of the heart rate and the blood pressure.

The trachea was intubated and the lung was mechanically ventilated via an endotracheal tube (inner diameter, 8.0 mm) in constant-volume mode (AVEA Ventilator; VIASYS Healthcare, Palm Springs, CA, USA): FiO_2 _of 0.4; positive end-expiratory pressure (PEEP) of 3 mbar; inspiratory to expiratory ratio of 1:1; tidal volume of 12 ml/kg; respiratory rate (RR) was set to maintain normocapnia. Ringer's solution at a rate of 5 ml/kg/hour was given throughout the entire experiment and was not changed. Before the lung lavage procedure started, hydroxyethyl starch (15 ml/kg; HES 130/0.4 Voluven^®^; Fresenius Kabi GmbH, Bad Homburg, Germany) was intravenously infused over 30 minutes. No further fluid boluses were applied during the experiment.

After exposure of the femoral vessels, a left ventricular catheter, an arterial catheter, a central venous line, and a pulmonary artery catheter with continuous cardiac output measurement (7.5 F Edwards CCO catheter connected to Edwards Vigilance CCO Monitor; Edwards Lifesciences Corp., Irvine, CA, USA) were inserted. The electrocardiogram, intravascular pressures, and left ventricular pressure were monitored continuously (S/5 Monitoring; Datex-Ohmeda, Duisburg, Germany). An aortic catheter was inserted via the left axillary artery for blood withdrawal during microsphere application, for intermittent arterial blood gas analysis (ABL 500; Radiometer, Copenhagen, Denmark), for arterial oxygen saturation, for determination of hemoglobin concentration (OSM 3 calibrated for swine blood; Radiometer), and for calibration of the continuous blood gas monitoring sensor (inserted via the femoral artery catheter, Paratrend 7; Diametrics Medical, High Wycombe, UK. The positions of the left ventricular catheter and pulmonary artery catheter were verified by typical waveforms.

All intravascular catheters were zeroed to the atmosphere. The midpoint between the anterior and posterior chest walls was taken as the zero reference point for pressure measurements. The animals were positioned in the prone position and a catheter was inserted into the right cerebral ventricle and connected to a fluid-filled pressure transducer (referenced to the meatus acusticus externus). All animals were thereafter placed in a supine position for the entire experiment. The distance between the mouth and the middle of the sternum was measured and marked on an esophageal catheter (SmartCath^® ^Esophageal catheter; VIASYS Healthcare) with an inflatable balloon at its tip. This catheter was connected to the esophageal pressure port of the ventilator (AVEA Comprehensive; VIASYS Healthcare), and an automated self-test (leakage test) and zeroing procedure (reference = atmosphere) was performed by the ventilator. The esophageal catheter was then inserted up to the marked position into the esophagus. The continuous measurement of the mean esophageal pressures started after activation of the software program of the ventilator and automated inflation of the balloon catheter with 0.5–1.25 ml air.

### Experimental protocol

Acute lung injury was induced by repetitive lung lavages until a PaO_2_/FiO_2 _ratio less than 13.3 kPa was achieved. The endotracheal tube was disconnected from the ventilator and isotonic Ringer's solution (20 ml/kg, 38°C) was instilled from a height of 70 cm above the endotracheal tube. After 30 seconds of apnea the fluid was retrieved by gravity drainage followed by endotracheal suctioning. After lung lavage, lung injury was progressed by ventilating the animals with a constant-volume mode and a PEEP of 5 mbar for 2 hours (FiO_2 _of 1.0; tidal volume of 12 ml/kg; inspiratory time of (T_insp_) 50% of the respiratory cycle; RR was set to achieve normocapnia). A continuous infusion of epinephrine was administered to maintain the mean arterial pressure between 70 and 80 mmHg during lung lavages and during the following two hours of mechanical ventilation. The administration of epinephrine and the infusion of Ringer's solution during the rest of the experiment were then kept constant.

After two hours, and in randomized order, a lung recruitment procedure was performed first by HFOV or by PCV. This was realized by a *P*_mean _step-up maneuver of 5 mbar every 30 minutes from 20 to 30 mbar. Every increase of *P*_mean _was performed slowly over 30 seconds. To achieve standardized conditions between HFOV and PCV, the endotracheal tube was disconnected for 30 seconds and mechanical ventilation was than re-established for 30 minutes (volume controlled ventilation; FiO_2 _of 1.0; PEEP of 5 mbar; inspiratory to expiratory ratio of 1:1; tidal volume of 12 ml/kg; RR was set to maintain normocapnia) before the subsequent respiratory mode (either HFOV or PCV) was performed.

**Figure 1:**
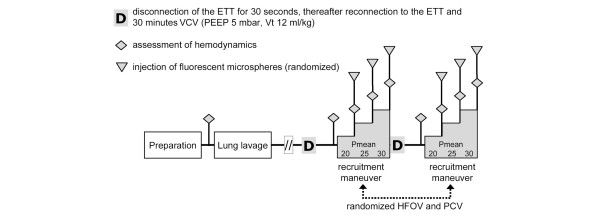
**Illustration of the study protocol**. ETT, endotracheal tube; HFOV, high-frequency oscillatory ventilation; PCV, pressure-controlled ventilation; PEEP, positive end-expiratory pressure; Pmean, mean airway pressure; VCV, volume-controlled ventilation; Vt, tidal volume.

During HFOV (High Frequency Oscillator Ventilator 3100b; Sensor Medics, Yorba Linda, CA, USA) the CDP (= *P*_mean_) was increased in steps of 5 mbar from 20, to 25 and 30 mbar every 30 minutes. The bias flow was set to 30 l/minute, the oscillatory frequency to 5 Hz, and the inspiratory time to 33% of the respiratory cycle. During PCV (AVEA Ventilator; VIASYS Healthcare) the *P*_mean _was increased from 20 to 25 to 30 mbar by increases of PEEP from 10 to 15 to 20 mbar, coupled to a constant inspiratory pressure amplitude (PEEP + 20 mbar) and an inspiration time of 50% of the respiratory cycle. The FiO_2 _was set to 1.0 with both ventilatory modes, and P_a_CO_2 _was maintained between 4.9 and 5.7 kPa by adjustment of the oscillatory pressure amplitude during HFOV and of the RR during PCV (see Figure 1).

### Measurements

All measurements were performed either during ongoing HFOV or during ongoing PCV. Thirty minutes after mechanical ventilation at each *P*_mean _setting (20, 25, or 30 mbar), the heart rate, mean arterial pressure, left ventricular end-diastolic pressure, central venous pressure, mean pulmonary artery pressure, pulmonary artery occlusion pressure, intracranial pressure, arterial hemoglobin, arterial and mixed venous blood gases, cardiac output (CO), mean esophageal pressure, and organ blood flows were obtained.

Adequate transmission of pleural pressures to the esophageal balloon catheter was verified by an occlusion test. This test was performed by moderately squeezing the chest and the abdomen while the airway was blocked, either after an inspiration or after an expiration. The position of the esophageal catheter was optimized to obtain a ratio of delta airway pressure/delta esophageal pressure of approximately 1 during thoracoabdominal compression maneuvers with the closed respiratory system [9].

The cardiac output was measured by the continuous thermodilution cardiac output technique (Edwards Vigilance CCO Monitor; Edwards Lifesciences Corp.). The 'STAT-Mode' of the Edwards Vigilance CCO Monitor was used in each experiment, which displayed the actual cardiac output values determined within the past 60 seconds. The last five measurements of CO were used and averaged. Numeric displayed values of intravascular pressures were recorded every 10 s for 1 minute during ongoing ventilation by PCV and HFOV with a switched off end-expiratory filter function of the monitoring system (S/5 Monitoring; Datex-Ohmeda).

The left ventricular end-diastolic pressure and pulmonary artery occlusion pressure were obtained as follows. The balloon of the pulmonary artery catheter was inflated and the monitor sweep was stopped. A vertical cursor was then adjusted to lie at the R-wave of the electrocardiogram and the left ventricular end-diastolic pressure was obtained from the indicated value from the left ventricular pressure wave, and the pulmonary artery occlusion pressure was obtained from the indicated value of the pulmonary artery catheter wave. This procedure was performed at three consecutive R-waves and three times regardless of the respiratory cycle.

All hemodynamic and ventilatory parameters were stored in a database sheet (Microsoft^® ^Excel 2002; Microsoft Corporation, Redmond, Washington, USA).

Organ blood flows were measured by the fluorescent microsphere technique, which is a validated method and is explained in detail elsewhere [10-13]. The general steps involved are: injection of a microsphere suspension into the animal circulation; isolation of organs and dissection into tissue volume elements; alkaline digestion of the solid tissue of each volume element to produce a tissue hydrolysate; centrifugation of the hydrolysate to isolate microspheres; solvation of microspheres to extract fluorescent dye; and measurement of the solution's fluorescence in different spectral regions with a spectrofluorometer. About two million microspheres were injected into the left ventricular catheter (six different colors, one for each measurement). The calculation of absolute blood flow rates was performed by reference blood sampling from the aortic catheter using a withdrawal pump (2 ml/minute).

At the end of each experiment the animals were euthanized (according to the recommendations of the *Report of the American Veterinary Medicine Association Panel on Euthanasia*) and the correct position of all catheters was verified by autopsy. The brains, hearts, kidneys and a jejunal section (10 cm) were removed and weighed. The microspheres were recovered from the tissue and from the blood by a sedimentation method [13,14].

Blood flows were calculated according to the formula: blood flow (ml/minute) = *I*_S _× *R *(ml/minute) × *I*_R_^-1 ^(where *I*_S _is the fluorescence intensity of sample, *I*_R _is the fluorescence intensity in the reference blood sample, and *R *is the reference withdrawal rate).

The transpulmonary pressure was calculated at each *P*_mean _setting during HFOV and PCV according to the formula: *P*_T _= *P*_mean _- mean esophageal pressure.

The pulmonary shunt (*Q*_s_/*Q*_t_) was calculated using a standard formula: *Q*_s_/*Q*_t _= Cc'O_2 _- CaO_2_/Cc'O_2 _- CvO_2 _(where *Q*_s _is the shunt flow, *Q*_t _is the cardiac output, and Cc'O_2_, CaO_2_, and CvO_2 _represent the oxygen content of pulmonary end-capillary, arterial and mixed venous blood, respectively). The oxygen contents of arterial (CaO_2_), mixed venous (CvO_2_) and pulmonary capillary (Cc'O_2_) samples were calculated using the following formula: content of oxygen = (hemoglobin concentration × 1.34 × percentage oxygen saturation/100) + (partial oxygen tension × 0.0031). To calculate Cc'O_2_, the pulmonary capillary oxygen tension was assumed to be equivalent to the alveolar partial oxygen tension, which was estimated as follows: FiO_2 _× (barometric pressure - water vapor pressure) - PaCO_2_/respiratory quotient. The value for the water vapor pressure was 47 mmHg and we assumed that the respiratory quotient was 0.8.

Oxygen delivery (DO_2_) was calculated according to the formula: DO_2 _= CO × CaO_2_.

The cerebral perfusion pressure was calculated as follows: cerebral perfusion pressure = mean arterial pressure - intracranial pressure.

### Statistical analysis

Data are expressed as the mean ± standard deviation. In each animal both the sequence of the two ventilatory modes (at first HFOV and secondly PCV, or at first PCV and secondly HFOV) and the order of the six different colors of microspheres were randomized by statistical software (BIASR Version 7.40; Epsilon-Verlag, Hochheim-Darmstadt, Germany) from a nonparticipant before the investigation started. The order of the *P*_mean _settings for lung recruitment were not randomized (the fixed sequence started at 20 mbar, increased to 25 mbar, and increased to 30 mbar every for 30 minutes).

An equal distribution for all data was analyzed by the Kolmogorov-Smirnov test. Differences for hemodynamics and blood gases before lung lavage and after lung lavage before HFOV and PCV were tested by paired *t *test. Analysis of variance for multiple measurements and pairwise multiple comparison procedures (Bonferroni *t *test) (Sigma Stat, Version 2.03; SPSS Inc., San Raphael, CA, USA) were used to evaluate the change of hemodynamics, ventilatory parameters, arterial blood gases, pulmonary shunt, and organ blood flows over time during HFOV and PCV, and to evaluate the differences of hemodynamics, ventilatory parameters, arterial blood gases, pulmonary shunt, and organ blood flows between the ventilatory modes (HFOV and PCV). Linear correlation analysis was performed to evaluate the association between the transpulmonary pressure and hemodynamics and between the right and left renal blood flow. Differences were considered statistically significant if *P *< 0.05.

## Results

The protocol was completed in all seven animals. Lung injury was induced by an average number of 4.1 ± 0.4 lung lavages (lavage volume, 2071 ± 189 ml). Epinephrine was administered at a rate of 0.04 (0.02–0.06) μg/kg/minute to maintain a mean arterial pressure between 70 and 80 mmHg during lung lavages and the following two hours of volume controlled ventilation. The mean volume of intravenously infused fluid volume was 1329 ± 122 ml during the experiment (mean duration, 7.5 ± 0.6 hours). No fluid boluses were applied during PCV and HFOV. Table 1 presents the ventilatory parameters, hemodynamics, and blood gas analysis before and after induction of lung injury. No differences in gas exchange and hemodynamics were noted before initiation of either HFOV or of PCV.

### Hemodynamics and blood flows

**Figure 2:**
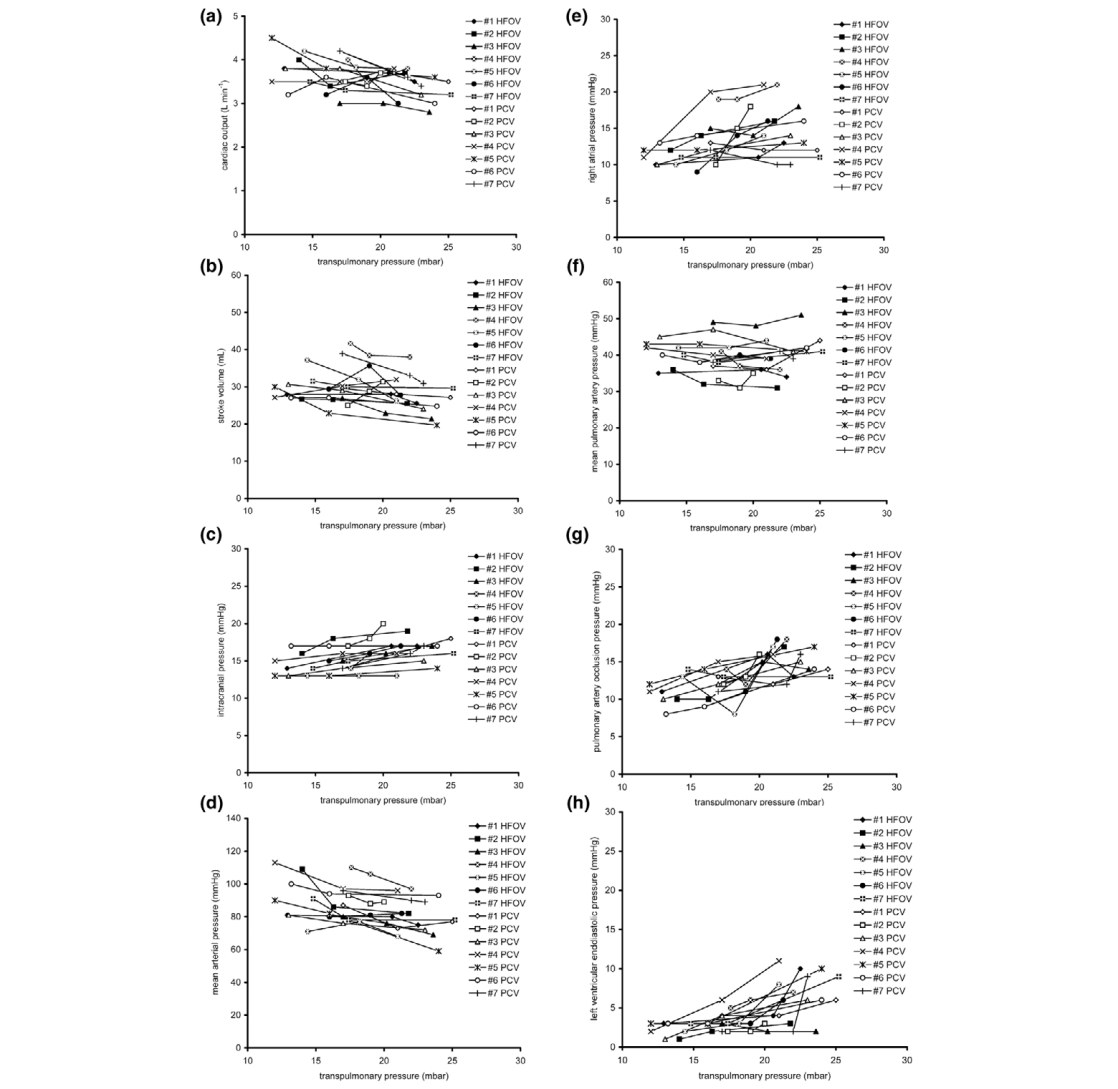
**Individual relationships between hemodynamics against corresponding transpulmonary pressures during high-frequency oscillatory ventilation and pressure-controlled ventilation**. Relationships during high-frequency oscillatory ventilation (HFOV) (filled symbols) and pressure-controlled ventilation (PCV) (open symbols) for **(a) **cardiac output, **(b) **stroke volume, **(c) **intracranial pressure, **(d) **mean arterial pressure, **(e) **right atrial pressure, **(f) **mean pulmonary artery pressure, **(g) **pulmonary artery occlusion pressure, and **(h) **left ventricular end-diastolic pressure. Animals are indicated #1–#7.

The results of the hemodynamic measurements for PCV versus HFOV are presented in Table 2 and Figure 2, where individual values of hemodynamics to corresponding transpulmonary pressures are graphically displayed. Measurements did not differ between both ventilation modes.

The elevation of *P*_mean _from 20 to 30 mbar lead to an increase of the heart rate (Table 2), right atrial pressure (HFOV, from 12 ± 4 mmHg to 15 ± 3 mmHg, *P *< 0.05; PCV, from 12 ± 2 mmHg to 16 ± 4 mmHg, *P *< 0.05), pulmonary artery occlusion pressure (HFOV, from 12 ± 2 mmHg to 16 ± 2 mmHg, *P *< 0.05; PCV, from 13 ± 2 mmHg to 15 ± 2 mmHg, *P *< 0.05), left ventricular end-diastolic pressure (HFOV, from 3 ± 1 mmHg to 6 ± 3 mmHg, *P *< 0.05; PCV, from 2 ± 1 mmHg to 7 ± 3 mmHg, *P *< 0.05), and intracranial pressure (HFOV, from 14 ± 2 mmHg to 16 ± 2 mmHg, *P *< 0.05; PCV, from 15 ± 3 mmHg to 17 ± 2 mmHg, *P *< 0.05) during HFOV and PCV. At the highest *P*_mean _setting of 30 mbar, the mean arterial pressure (HFOV, from 89 ± 7 mmHg to 79 ± 9 mmHg, *P *< 0.05; PCV, from 91 ± 8 mmHg to 81 ± 8 mmHg, *P *< 0.05), cerebral perfusion pressure (Table 2), cardiac output (HFOV, from 3.9 ± 0.4 l/minute to 3.5 ± 0.3 l/minute, *P *< 0.05; PCV, from 3.8 ± 0.6 l/minute to 3.4 ± 0.3 l/minute, *P *< 0.05), and stroke volume (HFOV, from 32 ± 7 ml to 28 ± 5 ml, *P *< 0.05; PCV, from 31 ± 2 ml to 26 ± 4 ml, *P *< 0.05) decreased during HFOV and PCV when compared with measurements at *P*_mean _levels of 20 mbar. The mean pulmonary artery pressure remained stable during all *P*_mean _variations at both ventilation modes.

The results of the linear correlation analysis between hemodynamics and transpulmonary pressure are presented in Table 3. The results of blood flow measurements are presented in Table 4. A homogeneous distribution of microspheres to the organs was indicated by significant linear correlation (*r *= 0.98, *r*^2 ^= 0.95, *P *< 0.000001, confidence interval (*P *= 0.99) = 0.91–0.99) between the blood flow of the right kidney (271 ± 131 ml/100 g/minute) and of the left kidney (270 ± 128 ml/100 g/minute). There were no differences between left and right renal blood flow. The left ventricular and right ventricular blood flow did not vary during *P*_mean _variations. Renal blood flow did not change during increases of *P*_mean _and showed no differences between HFOV and PCV. Jejunal blood flow showed no deterioration during airway pressure increases. Also, the cerebral blood flow in the hemispheres, the cerebellum and the brainstem was not influenced by different *P*_mean _levels and showed no differences between HFOV and PCV.

### Transpulmonary pressure, pulmonary gas exchange, and pulmonary shunt

All ventilatory parameters, PaO_2 _and PaCO_2_, and calculated pulmonary shunt fraction data are presented in Table 2. The *P*_T _increased at every *P*_mean _level during HFOV and PCV, and was comparable between both ventilatory modes at each *P*_mean _setting. Oxygenation improved after initiation of HFOV and PCV by a stepwise increase of *P*_mean_, starting at 20 mbar, followed by 25 and 30 mbar. To maintain normocapnia at a *P*_mean _level of 30 mbar, increased oscillatory pressure amplitudes (Table 2) during HFOV and increased respiratory rates during PCV were necessary. The *P*_mean _of 30 mbar during PCV was accompanied by lower tidal volumes and decreased dynamic compliance of the respiratory system.

Measurement of tidal volumes and dynamic compliance of the respiratory system during HFOV was technically not possible. At similar *P*_mean _levels, the PaO_2 _and PaCO_2 _values showed no differences between HFOV and PCV. As shown in Table 4, pulmonary shunt values decreased to physiological values (less than 5%) at the highest *P*_mean _setting in all animals, whereas at a *P*_mean _level of 25 mbar the pulmonary shunt was reduced by HFOV only. Oxygen delivery was unchanged when *P*_mean _increased, independent of the ventilatory mode used (Table 2).

## Discussion

Lung recruitment procedures by incremental increases of lung volumes and airway pressures may impair hemodynamics and organ blood flow [15,16]. The present study compared a typical recruitment maneuver up to a *P*_mean _of 30 mbar by HFOV with a recruitment maneuver by PCV at similar *P*_mean _settings in a lung lavage model. The lung lavage model affects particularly the lung, whereas other organs are not involved, and organ blood flow autoregulation is theoretically intact. In this setting, we observed decreases of the arterial pressure, cardiac output, and stroke volume, and observed increases of the heart rate, central venous pressure, pulmonary artery occlusion pressure, left ventricular end-diastolic pressure, and intracranial pressure during lung recruitment in both ventilatory modes. The cerebral blood flow, myocardial blood flow, renal blood flow, and blood flow of the jejunum, however, were not reduced during stepwise increases of the mean airway pressure up to 30 mbar in the lung-injured animals. Transpulmonary pressures during HFOV and PCV were comparable. Organ blood flow and systemic hemodynamics did not differ between both ventilatory modes. These results may differ in a scenario without inotrope and vasoactive drug administration or when extrapulmonary organ dysfunctions are present (e.g. sepsis, septic shock, intracranial pathology, or multiple organ failure).

Transition to HFOV requires a recruitment procedure of the lung at initiation, typically performed by slow stepwise increases of continuous distending pressure to optimize the alveolar volume available for gas exchange, as used in several clinical studies [5-8]. This procedure differs from recruitment maneuvers by conventional ventilation modes, which use sustained or intermittent PEEP or inspiratory pressure level increases (such as, deep lung inflation of various magnitudes and durations). During HFOV, the expansion of the lung and chest wall continues constantly without excursions related to large tidal volume or airway pressure when compared with conventional low-frequency ventilation modes [17]. The cardiovascular effects of increasing intrathoracic pressures during low-frequency positive-pressure ventilation are well investigated. The portion of the applied intraalveolar pressure transmitted across the lung (transpulmonary pressure) may rise at higher *P*_mean _but depends mainly on the elastance of the chest wall and the lung [18]. High transpulmonary pressures have been associated with increases in cardiac filling pressures, and decreases in venous return, cardiac output, and arterial pressures [3,4].

The right ventricular afterload may increase when high airway pressures are applied and subsequent right ventricular enlargement could alter the left ventricular performance by ventricular interdependence (that is to say, leftward shift of the ventricular septum with decreased left ventricular compliance and disturbance of septal wall motion). Also, an increased lung volume with exhausted compensation mechanisms (descendent diaphragm, expanded rib cage) during lung recruitment can affect cardiac function and hemodynamics by direct mechanical compression of the heart into the cardiac fossa. Experimental and clinical studies have demonstrated effects upon hemodynamics with initiation of HFOV at high mean airway pressures, whereas other studies did not find this effect [5-8,19-23].

In the literature, HFOV has been associated with a decrease in arterial pressures, cardiac output, and stroke volume because of reduced venous return. Systemic hemodynamics decreased during lung recruitment maneuvers by HFOV and PCV, but remained in the normal ranges in the present study; it is expected that these effects can easily corrected either by volume administration or by the adaptation of the vasoactive drug dosage. One possible explanation for the impairment in the hemodynamics is right ventricular dysfunction due to an increased impedance to the right ventricular output, resulting in dilatation of the right ventricle, in displacement of the interventricular septum towards the left ventricle, and hence in impairment of left ventricular filling. We did not, however, observe any signs of severe right heart dysfunction during increases of *P*_mean _and *P*_T_.

The magnitude of effects upon the cerebral perfusion pressure and the intracranial pressure was minor in animals without intracranial pathology but with an unchanged administration of epinephrine. All recorded hemodynamic effects were comparable at similar *P*_T _levels between PCV and HFOV. In this setting, therefore, the *P*_T _level that interacts with the cardiorespiratory unit is the main determinant of hemodynamic response, and not the used ventilatory mode. The used PCV settings for lung recruitment, however, did not incorporate the recommended ventilatory strategy in humans with acute lung injury and acute respiratory distress syndrome (tidal volume, 6 ml/kg predicted bodyweight; inspiratory pressure limitation, 35 mbar; permissive hypercapnia), and it is well known that inspiratory inflation at high lung volumes may limit cardiac volumes. Normocapnia was maintained during HFOV and PCV to exclude a significant source of bias in respect to substantial hypercapnia-associated effects upon hemodynamics and organ blood flow [24,25].

In this scenario, the blood flow to the brain, heart, kidneys, and jejunum was unaffected when *P*_mean _and *P*_T _increased. This may be due to the absence of severe effects of the increased *P*_T _upon systemic hemodynamics and due to the fact that blood flow autoregulation of organs was still intact because of only one organ failure (lung injury induced by lung lavage).

With respect to short-time effects, Nunes and colleagues reported in healthy pigs impaired intestinal blood flows within minutes at high airway pressures (continuous positive airway pressure of 40 mbar for 20 seconds), but these effects recovered quickly after the lung recruitment procedure [26]. Dorinsky and colleagues reported decreased CO, but unaffected regional blood flow (kidneys, heart, brain) at high PEEP levels (25 mbar) after 30 and 60 minutes in healthy pigs [27].

The effects of elevated airway pressures and the resulting transpulmonary pressures upon different vascular beds and organ perfusion, however, may be more pronounced in a clinical situation with acute lung injury/acute respiratory distress syndrome, concomitant extrapulmonary organ dysfunction, and impaired tissue perfusion. Oxygenation improved during HFOV and PCV without differences between both ventilatory modes at high mean airway pressures. The calculated pulmonary shunt fraction (that is to say, venous admixture) fulfilled the criteria (pulmonary shunt less than 10%) of complete reopened lungs [28]. Simultaneously, the recruitment of closed alveolar units was paralleled by pulmonary hyperinflation, indicated by decreased CO_2 _clearance because of increased dead space when *P*_mean _was set to 30 mbar. The oscillatory pressure amplitude during HFOV and the RR during PCV had to be increased to maintain the arterial PCO_2 _in the predefined range.

## Limitations

The present study is experimental and the results cannot directly be extrapolated to patients with lung injury and without use of inotropic drug and vasoactive drug administration. The used method for blood flow measurement allowed only a single assessment at each *P*_mean _setting (one measurement 30 minutes after each *P*_mean _adjustment), and negative effects before this measurement as well as long-lasting effects cannot be excluded. The resulting tidal volumes during lung recruitment procedures by PCV were higher than the recommended tidal volume of 6 ml/kg predicted bodyweight in humans with acute lung injury or acute respiratory distress syndrome. The findings of an HFOV initiation protocol by stepwise increases of CDP can therefore only be compared with the used lung recruitment strategy by PCV with PEEP increases coupled to a constant inspiratory pressure amplitude (PEEP + 20 mbar). According to the randomization, HFOV was used as the second mode in four animals whereas only three animals received PCV as the second mode. Recovery from lavage-induced lung injury over time by endogenous production of surfactant cannot be excluded. Hence, a bias of the results due to a time effect cannot be excluded and might have favored one group.

## Conclusion

The present experimental study in lung-injured pigs with unchanged dosages of a positive intotrope and a vasoactive drug demonstrates that a typical lung recruitment maneuver as used clinically at initiation of HFOV decreases the systemic hemodynamics, improves oxygenation, decreases pulmonary shunt, but has no negative influence upon blood flow to the brain, the kidneys, the jejunum and the heart. The stabilization of organ blood flows may be due to the absence of severe changes of systemic hemodynamics in lung-injured pigs and the assumption that blood flow autoregulation of organs was intact. Changes of macrohemodynamics were dependent on the transpulmonary pressure level, however, and were not associated with HFOV *per se*. All effects were similar to the used settings of conventional low-frequency PCV at comparable transpulmonary pressures. The effects of HFOV-associated effects upon organ perfusion in a scenario with acute lung injury and concomitant multiple organ failure need to be addressed in further studies.

## Key messages

• 	A lung recruitment maneuver by stepwise increases of the mean airway pressure to 30 mbar either by PCV with tidal volumes of 10–13 ml/kg or by HFOV had similar effects on cardiac performance and on blood flow to the nonpulmonary organs.

• 	The results of this study cannot be extrapolated to clinical situations without the use of inotropic drugs or vasoactive drugs.

## Abbreviations

CDP = continuous distending pressure; CO = cardiac output; FiO_2 _= inspiratory oxygen fraction; HFOV = high-frequency oscillatory ventilation; PaCO_2 _= arterial partial pressure of carbon dioxide; PaO_2 _= arterial partial pressure of oxygen; PCV = pressure-controlled ventilation; PEEP = positive end-expiratory pressure; *P*_mean _= mean airway pressure; *P*_T _= transpulmonary pressure; *Q*_s_/*Q*_t _= pulmonary shunt; RR = respiratory rate.

## Competing interests

The authors declare that they have no competing interests.

## Authors' contributions

MD and KM initiated the study, the design and the experimental protocol. MD, HWG, JK, and ALD conducted the experiments and the analysis of fluorescent microspheres for organ blood flow measurements. OK supported the analysis of microspheres. MD and KM performed the statistical analysis. MD wrote the manuscript, and KM and OK helped to draft the manuscript. All authors read and approved the final manuscript.

## Figures and Tables

**Table 1:** Ventilatory parameters, hemodynamics, and blood gas analysis before and after induction of lung injury

	Healthy animal	Lung lavage before PCV	Lung lavage before HFOV
Plateau airway pressure (mbar)	20 ± 2	33* ± 2	34* ± 3
Mean airway pressure (mbar)	9 ± 1	13* ± 2	13* ± 2
Static lung compliance (ml/mbar)	21 ± 1	11* ± 1	10* ± 1
Respiratory rate (minute^-1^)	16 ± 2	16 ± 2	16 ± 2
tidal volume per kg bodyweight (ml/kg)	12.8 ± 0.8	12.1 ± 0.2	12.3 ± 0.2
expiratory minute ventilation (l/minute)	4.7 ± 0.7	5.0 ± 0.6	4.9 ± 0.6
Heart rate (minute^-1^)	112 ± 12	127* ± 25	125* ± 18
Mean arterial pressure (mmHg)	80 ± 11	81 ± 6	81 ± 8
Right atrial pressure (mmHg)	13 ± 2	12 ± 3	12 ± 2
Mean pulmonary arterial pressure (mmHg)	26 ± 6	39* ± 6	40* ± 6
Pulmonary artery occlusion pressure (mmHg)	10 ± 3	13 ± 5	14 ± 3
Left ventricular end-diastolic pressure (mmHg)	2 ± 1	3 ± 1	3 ± 1
Intracranial pressure (mmHg)	11 ± 2	13 ± 2	13 ± 1
Cardiac output (l/minute)	3.3 ± 0.3	3.8 ± 0.5	3.7 ± 0.6
Stroke volume (ml)	28 ± 4	29 ± 8	30 ± 6
PaO_2 _(kPa)	65.9 ± 8.9	10.8* ± 1.7	11.3* ± 1.9
PaCO_2 _(kPa)	5.5 ± 0.3	5.5 ± 0.4	5.6 ± 0.3
Pulmonary shunt (%)	3 ± 1	38* ± 4	39* ± 9

Measurements taken during volume-controlled ventilation (positive end-expiratory pressure, 5 mbar; FiO_2_, 1.0). No differences were found between lung-injured animals before transition to either high-frequency oscillatory ventilation (HFOV) or pressure-controlled ventilation (PCV). Data presented as the mean ± standard deviation. Static lung compliance = tidal volume/(plateau airway pressure - positive end-expiratory pressure). *P < 0.01 versus healthy lungs.

**Table 2:** Transpulmonary pressures, ventilatory parameters, arterial blood gases, calculated pulmonary shunt, oxygen delivery, heart rate, and cerebral perfusion pressure during a lung recruitment procedure by successive increases of mean airway pressure

	20 mbar mean airway pressure		25 mbar mean airway pressure		30 mbar mean airway pressure	
	
	HFOV	PCV	HFOV	PCV	HFOV	PCV
Transpulmonary pressure (mbar)	15 ± 3	15 ± 3	19^c ^± 2	18^a ^± 3	22^cd ^± 2	23^ab ^± 2
Respiratory rate (minute^-1^)	300	18 ± 10	300	21 ± 11	300	27^ab ^± 10
Oscillatory pressure amplitude (mbar)	40 ± 7	NA	41 ± 8	NA	52^cd ^± 8	NA
Dynamic compliance of the respiratory system (ml/mbar)	NA	18 ± 5	NA	17 ± 4	NA	12^ab ^± 3
Tidal volume per kg bodyweight (ml/kg)	NA	13 ± 3	NA	12 ± 4	NA	10^ab ^± 2
PaO_2 _(kPa)	21 ± 4	19 ± 6	57^c ^± 10	43^a ^± 21	69^cd ^± 7	71^ab ^± 11
PaCO_2 _(kPa)	5.3 ± 0.3	5.4 ± 0.3	5.4 ± 0.31	5.3 ± 0.3	5.4 ± 0.3	5.4 ± 0.3
Pulmonary shunt (%)	22 ± 8	23 ± 7	6^c ^± 3	10 ± 6	3^cd ^± 1	3^a ^± 1
Oxygen delivery (ml/minute)	347 ± 64	356 ± 73	341 ± 65	353 ± 50	335 ± 63	338 ± 57
Heart rate (minute^-1^)	119 ± 16	123 ± 19	121 ± 16	129 ± 19	129^b ^± 18	134^a ^± 18
Cerebral perfusion pressure (mmHg)	74 ± 15	80 ± 10	68 ± 10	70 ± 8	62^b ^± 9	65^a ^± 13

Data presented as the mean ± standard deviation. HFOV, high-frequency oscillatory ventilation; PCV, pressure-controlled ventilation. Dynamic compliance of the respiratory system = tidal volume/(endinspiratory pressure - positive end-expiratory pressure). ^a^*P *< 0.05 compared with PCV 20 mbar, ^b^*P *< 0.05 compared with PCV 25 mbar, ^c^*P *< 0.05 compared with HFOV 20 mbar, ^d^*P *< 0.05 compared with HFOV 25 mbar. NA, not applicable.

**Table 3:** Linear correlation analysis between transpulmonary pressure and hemodynamics during a lung recruitment procedure by successive increases of mean airway pressure

	High-frequency oscillatory ventilation	Pressure-controlled ventilation
Cardiac output	-0.29 (0.18)	-0. 53 (0.01)
Stroke volume	-0.23 (0.33)	-0.18 (0.41)
Intracranial pressure	0.48 (0.03)	0.42 (0.06)
Mean arterial pressure	-0.46 (0.04)	-0.52 (0.01)
Right atrial pressure	0.44 (0.04)	0.19 (0.42)
Mean pulmonary artery pressure	0.17 (0.45)	-0.10 (0.65)
Pulmonary artery occlusion pressure	0.52 (<0.01)	0.67 (<0.01)
Left ventricular end-diastolic pressure	0.57 (0.04)	0.63 (0.002)

Data presented as correlation coefficient (*P *value).

**Table 4:** Organ blood flows (ml/100 g/min) during a lung recruitment procedure by successive increases of mean airway pressure

	20 mbar mean airway pressure		25 mbar mean airway pressure		30 mbar mean airway pressure	
	
	HFOV	PCV	HFOV	HFOV	PCV	HFOV
Left ventricle	220 ± 115	239 ± 74	240 ± 100	266 ± 64	191 ± 55	197 ± 79
Right ventricle	163 ± 72	208 ± 80	206 ± 88	221 ± 66	172 ± 86	209 ± 80
Kidneys	293 ± 89	265 ± 88	258 ± 102	298 ± 80	276 ± 102	240 ± 37
						
Jejunum	47 ± 22	44 ± 22	47 ± 19	53 ± 17	48 ± 25	47 ± 17
Hemispheres	55 ± 23	53 ± 20	46 ± 22	53 ± 18	45 ± 23	47 ± 10
Cerebellum	43 ± 18	49 ± 16	46 ± 16	48 ± 22	46 ± 15	51 ± 10
Brainstem	40 ± 19	46 ± 13	36 ± 14	43 ± 13	37 ± 19	39 ± 17

Organ blood flow was unchanged when the mean airway pressure increased and no differences were found between high-frequency oscillatory ventilation (HFOV) and pressure-controlled ventilation (PCV). Data presented as the mean ± standard deviation.
